# Addressing Social Determinants of Health by Integrating Assessment of Caregiver-Child Attachment into Community Based Primary Health Care in Urban Kenya

**DOI:** 10.3390/ijerph9103588

**Published:** 2012-10-12

**Authors:** John H. Bryant, Nancy H. Bryant, Susanna Williams, Racheal Nduku Ndambuki, Paul Campbell Erwin

**Affiliations:** 1 Orphans and Vulnerable Children’s Project in Africa, and Department of International Health, Johns Hopkins Bloomberg School of Public Health, and Department Public Health Sciences, University of Virginia Faculty of Medicine, 250 Pantops Mountain Road, Apt 5223 Charlottesville, VA 22911, USA; Email: jbryantwcbr@gmail.com; 2 Orphans and Vulnerable Children’s Project in Africa, 250 Pantops Mountain Road, Apt 5223 Charlottesville, VA 22911, USA; Email: nbryant1931@live.com; 3 Guatemala Initiative, School of Medicine, University of Virginia, 737 Locust Avenue Charlottesville, VA 22902, USA; Email: susanna2020@comcast.net; 4 Orphans and Vulnerable Children’s Project in Africa, P.O. Box 433-00242 Kitengela, Nairobi, Kenya; Email: oldmlolongohdept@yahoo.com; 5 Department of Public Health, University of Tennessee, 1914 Andy Holt Avenue, Knoxville, TN 37996, USA

**Keywords:** social determinants of health, early childhood development, caregiver-child attachment

## Abstract

A principle strategic insight of the Final Report for WHO’s Commission on Social Determinants of Health (SDOH) is that the nurturant qualities of the environments where children grow up, live, and learn matter the most for their development. A key determinant of early childhood development is the establishment of a secure attachment between a caregiver and child. We report initial field-tests of the integration of caregiver-child attachment assessment by community health workers (CHWs) as a routine component of Primary Health Care (PHC), focusing on households with children under 5 years of age in three slum communities near Nairobi, Kenya. Of the 2,560 children assessed from July–December 2010, 2,391 (90.2%) were assessed as having a secure attachment with a parent or other caregiver, while 259 (9.8%) were assessed as being at risk for having an insecure attachment. Parent workshops were provided as a primary intervention, with re-enforcement of teachings by CHWs on subsequent home visits. Reassessment of attachment by CHWs showed positive changes. Assessment of caregiver-child attachment in the setting of routine home visits by CHWs in a community-based PHC context is feasible and may yield valuable insights into household-level risks, a critical step for understanding and addressing the SDOH.

## 1. Introduction

A principle strategic insight of the Final Report for the World Health Organization’s (WHO’s) Commission on Social Determinants of Health is that the nurturant qualities of the environments where children grow up, live, and learn matter the most for their development [1]. In a recent review article Hertzman and Boyce elaborate on this process whereby environmental influences “get under the skin” and influence biological systems, a process described as “biological embedding” [2]. Advances in the science of early childhood development (ECD) provide powerful evidence that what a child experiences during the early years - this biological embedding—sets a critical foundation for the entire life-course: ECD—including physical, social/emotional and language/cognitive domains—strongly influences basic learning, school success, economic participation, social citizenry, and health [1,2,3,4]. One of the most important determinants of ECD—caregiver-child attachment—focuses on the importance of close, loving, and stimulating interactions of the young child and mother or caregiver from day one after birth and through the early weeks, months, and years, which can result in life-long benefits. Recent research has identified two fundamental qualities that determine the caregiver’s ability to provide effective care: sensitivity and responsiveness to the child [4]. These skills enable the caregiver to detect the child’s signals and to respond appropriately, in synchrony, to meet the child’s needs, promoting the development of a child who is physically, intellectually, and socially healthy and more resilient to the damaging effects of poverty, violence, and other social determinants of health. 

While community-based interventions and primary health care (PHC) systems often contain many elements related to ECD in general—including growth monitoring and immunizations—there are no published reports of incorporating assessment of caregiver-child attachment as a *routine *component of such programs [5,6]. The primary purpose of this article is to describe the development and initial findings of a community-based PHC program in slum communities near Nairobi, Kenya, which has included the assessment of caregiver-child attachment as a routine component of household visits by community health workers (CHWs).

### 1.1. Background

The “new science” in ECD supports much of the theoretical framework of attachment theory set forth by John Bowlby and further developed by Mary Ainsworth in the mid- to late-twentieth century [7]. Three recent primary documents provide much of the evidence-based underpinnings for the work described in this article. First, in *From Neurons to Neighborhoods—The Science of Early Childhood Development*, scientists take account of the fact that genetic and environmental influences work together in dynamic ways over the course of development [3]. The most important questions now concern how environments influence the expression of genes and how genetic makeup, combined with children’s previous experiences, affects their ongoing interactions with their environments during the early years and beyond.

The second important reference is the World Health Organization (WHO) publication *The importance of caregiver-child interactions for the survival and healthy development of young children. A Review* [4]. This review lays the groundwork for including interventions to improve the relationship between the caregiver and child in an overall strategy to improve the child’s survival, health, and development. 

A third major reference for guiding the work described in this article is *Early Childhood Development: A Powerful Equalizer, the Final Report for WHO’s Commission on Social Determinants of Health* [1]. As noted above, this document indicates that the qualities of the environments matter the most for child development; however, it goes on to emphasize that parents cannot provide strong nurturant environments without help from local, regional, national and international agencies. 

### 1.2. The Setting

This project—The Orphans and Vulnerable Children’s (OVC) Project in the Urban Slums of Africa—began in 2005 in response to a commitment made by UN Habitat to the Millennium Development Goal of improving the well-being of 100 million African slum dwellers. Concerned that the needs of young children might be left out, UN Habitat asked two of the authors (J. Bryant and N. Bryant) to help develop health care and social support for children under five years of age in the urban slums of Kenya. UN Habitat and the Kenya Slum Upgrading Project recommended three urban slums outside of Nairobi—Mlolongo, Sophia, and Bondeni—with a total estimated population of 215,000. This project was subsequently supported through a Rockefeller Foundation grant to the African Population and Health Research Center (APHRC) in Nairobi, as a comprehensive, multi-level project focused on addressing HIV/AIDS in Kenya. 

The Health Committees of these communities participated in the formulation of a Community-Based Primary Health Care (PHC) approach that included all children under 5 years of age. The OVC component of the project used a community empowerment approach in the targeted slum communities to develop two major programmatic activities, which remain operational. One is traditional, community-based PHC, including growth monitoring, immunizations, use of insecticide treated bed nets, hand washing, oral rehydration therapy, nutritional supplementation, and general child well-being. The second component includes assessment and monitoring of caregiver-child attachments, which is the focus of this current article. An important aspect of the project has been the selection and training of 24 CHWs, eight in each community, who provide these services. They are women and men from these communities, selected by the Health Committees, who agreed to visit every household on a part-time basis, maintaining records on elements of PHC and caregiver-child interactions. Each CHW was assigned a zone to work in that was close by to where he/she lived. The CHW was then assigned families with children under five in that zone; thus, households would then be assigned to the CHWs on the basis of their (CHWs) knowledge of and familiarity with that specific area of the slums. Twenty-four CHWs have been selected and trained, with each having a target of 100 households. Training was provided for CHWs and OVC staff on the basic components of PHC as well as the assessment of caregiver-child attachment. One author (R. Nduku), who lives in one of the slums, serves as Project Coordinator, overseeing all important aspects of the project including social support, computerized data collection, and oversight of the CHWs. 

## 2. Methods

**Figure 1:**
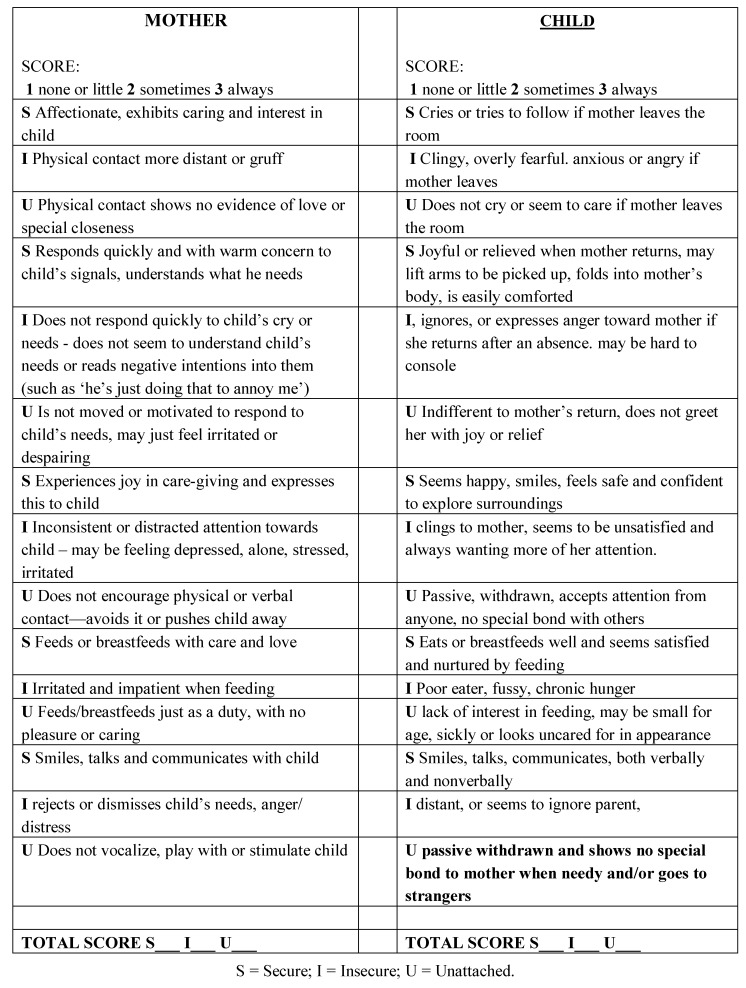
Attachment Assessment Score Sheet, derived from the African version of the Circle of Security (^©^ Robert Marvin [8]).

Households in the targeted communities with one or more children under five years of age were included in this project. Each CHW was assigned households in the specific area most familiar to the CHW, until the CHW reached a maximum of 100 assigned households, for a maximum total of 2,400 households. Regular visits to households with children under 5 years of age began in 2008, and included many components of typical PHC models that are used in similar settings. CHWs were assigned to visit each household at least once every three months. Children assessed as underweight (weight for age), using standard WHO growth charts, were provided a nutrition-rich millet porridge (*wimbi*) to supplement their daily intake. Caregivers of children with diarrhea (two or more watery, loose stools per day) were provided advice about handwashing, the use of clean water, and the use of oral rehydration. Children were assessed to have malaria if they were found to have a constellation of symptoms that may have included fever, vomiting, feeling cold, headache and shivering, muscles weakness, joint pains and/or convulsions, or if they were diagnosed with malaria in a nearby health centre. Households without bednets were provided a single insecticide-treated bednet to protect children in the household, and children assessed as acutely ill were referred to a local government-run health centre. A one-page instrument was designed for the CHWs to assess the quality of the caregiver-child relationship as they performed routine home visits (Figure 1). This scoring sheet was constructed with the guidance of Dr. Robert Marvin, Director of the Mary Ainsworth Attachment Clinic (University of Virginia, Charlottesville, VA, USA) and based on multiple tools used for evaluating parenting behavior as it relates to the attachment relationship with their child (including the Circle of Security Index [8], the Parent Development Interview [9], and the Adult Attachment Interview [10]). These instruments were adapted to the specific culture of the African community and informed the development of the attachment assessment score sheet (Figure 1) specifically for the OVC project.

The CHWs were trained to recognize key behavioral indicators and evaluate caregiver-child relationships in the context of the routine home visits. Teaching materials included key diagrams translated into Swahili and video tapes created by filming many community members in their homes, as they went through an adapted version of the Ainsworth Strange Situation [11], the classic controlled experiment which demonstrates attachment behavior. The tapes were edited to show examples of attachment behavior, as portrayed in their own cultural context, and used to train the CHWs to recognize and be able to code for these behaviors using the score sheet shown in Figure 1. 

The attachment assessment score sheet was used to identify behaviors *consistent *with secure and insecure attachment along a continuum, resulting in a numeric score indicating the nature of the relationship—essentially quantifying a qualitative element, but from a “risk assessment” perspective rather than a purely diagnostic one. A CHW made three home visits before determining the status of the relationship. 

An intervention program was developed for caregivers who were identified as being at risk for having insecure attachments. This program centered on parent workshops, and involved a follow-up program utilizing peer support, and further follow-up four months later as the CHW re-evaluated the relationship. This program was shaped around an evidence-based training program used in a therapeutic attachment clinic [12], which was adapted to the local culture and the CHWs’ education level. 

Research authorization was granted by the National Council for Science and Technology, Government of Kenya. The original study—which was focused on HIV/AIDS in the slum communities and was under the auspices of the APHRC—was approved by the Kenya Medical Research Institute’s ethical review committee, who are responsible for conducting such reviews at the national level. This manuscript reflects only one aspect of the original study, namely the PHC and attachment focus. In addition, a parallel project involved one of the authors (S. Williams) in research leading to a dissertation [13]; IRB approval for that research was provided by the University of Virginia.

Data from CHW visits were entered into Access and analyzed in Stata (version 10, copyright 1984–2008, StataCorp, College Station, TX, USA). Data analysis included basic descriptive statistics and chi-square for measures of association between categorical variables. 

## 3. Results

Usable data were provided for 2,560 children visited by CHWs between July-December 2010. A biological parent was documented to be the primary caregiver for 92% of the children. CHW assessment of caregiver-child attachment showed that 9.8% of the children were at risk of being insecurely attached to their primary caregiver. Attachment did not vary significantly by community or gender. Children assessed as at-risk for being insecure were much less likely than children with assessments consistent with secure attachment to regularly wash hands (*p* < 0.05) and to have a normal weight for age (*p* < 0.001) and more likely to have diarrhea and malaria (*p* < 0.001).

**Table 1:** Measures of, and associations with, attachment between caregivers and children <5 years of age at initial visit for three targeted communities, 2010.

Community	% At-risk for insecure attachment	% Consistent with secure attachment	
Bondeni (n = 940)	11.1	88.9	
Mlolongo (n = 1,032)	9.1	90.9	
Sophia (n = 678)	9.0	91.0	
Gender			
Female (n = 1,356)	10.3	89.7	
Male (n = 1,294)	9.2	90.8	
**Health Indicator**	**Insecure**	**Secure**	***p*, Chi-square**
Has Clinic Card			
No (n = 398)	4	394	<0.001
Yes (n = 2,252)	255	1,997	
Regular Handwashing			
No (n = 2,148)	225	1,923	<0.05
Yes (n = 502)	34	468	
Bednet present			
No (n = 1,785)	91	1,694	<0.001
Yes (n = 865)	168	697	
Has Diarrhea			
No (n = 2,012)	37	1,975	<0.001
Yes (n = 638)	222	416	
Has Malaria			
No (n = 2,390)	76	2,314	<0.001
Yes (n = 260)	183	77	
Normal weight for age			
No (n = 226)	256	0	<0.001
Yes (n = 2,394)	3	2,391	

Follow-up assessments after interventions were delivered showed that among the 259 children initially assessed as at-risk for being insecure, 215 (83%) were assessed as having a secure attachment (data not shown). 

## 4. Discussion

The key findings of this project to-date relate to the process of incorporating assessment of caregiver-child attachment within a community-based PHC system, the basic description of attachment levels in these slum communities, and initial findings related to field-based interventions to address insecure attachments. This may provide one means of addressing the SDOH where it matters most—at the level of interaction between child, caregiver, and their environments.

In contrast to our finding that ~90% of children <5 years had secure attachment to a caregiver, Kermoian and Leiderman reported that 61% of infants in a study in southwestern Kenya were assessed as securely attached to their mothers [14]. True *et al.* found that 67% of infant-mother dyads in the Dogon of Mali were assessed as secure [15], while Tomlinson *et al.* reported 62% of infants with secure attachment in a study in South Africa [16]. While these comparisons may be useful for framing the overall burden of insecure attachment, the methods used (modified Strange Situation) in deriving these estimates are very different from the CHWs’ use of the attachment assessment instrument. Numerous systematic reviews—most recently by Berlin *et al.* [17] —document the impact of a wide variety of interventions to ameliorate insecure attachment; however, no field-based interventions similar to the OVC project were noted. 

Research findings of one of the authors (S. Williams), who completed doctoral work with a group of young mothers in one of the slums (Mlolongo), indicated that most of them had secure attachments to their children [13]. Insecure attachment relationships seemed to occur most often in situations where the caregiver was suffering from depression, raising children who were not her own biological offspring (as in the case of AIDS orphans), encountering severe poverty, or had become pregnant as teens. Adolescent mothers were often overwhelmed, had no access to extended family for support, and were at high risk for chronic disease such as AIDS—all factors which were generally not conducive to developing secure attachments with their children.

There are no other identifiable published reports of incorporating caregiver-child attachment assessment as a *routine* component of PHC model systems. Although most attachment assessment methodologies have been developed within the Western context, Mary Ainsworth, considered by many to be the leading pioneer in attachment theory and research, conducted her initial work in Uganda [18]. Her foundational work led to the development of the (Ainsworth’s) Strange Situation procedure for assessing the security of attachment [11], and most forms of “laboratory-based” or controlled experimental methods for assessing attachment use the Strange Situation procedure as a foundation [19]. A method for assessing the quality of a child’s secure-base behavior in the home was developed by Everett Waters, known as the Attachment Q-Set measure (AQS) [20]. As noted by Solomon and George, “The great promise of AQS lies in its emphasis on naturalistic observation in ecologically valid contexts. Researchers have demonstrated that the procedure can be used reliably and with adequate validity across a variety of national and cultural groups” [19] (p. 406). Both the Strange Situation and the AQS have been applied in a wide array of settings outside of North America and Western Europe, including Israel, Japan, China, Indonesia, Puerto Rica, Mexico, and Africa [19]. Peterson *et al.* used the AQS to assess attachment in a sample of Ugandan women [21] to study the relationship of maternal and child HIV/AIDS infection to attachment, and Kermoian and Leiderman used a modified Strange Situation approach to study the differences in attachment between children and mothers *vs.* child caregivers in southwestern Kenya [14]. 

The limitations to the OVC’s activities and this report include: limited validation of the caregiver-child assessment tool for use within this specific socio-cultural setting; inability to specify nutritional deficiencies—such as iron deficiency anemia—which may have confounded the results of caregiver-child attachment; insufficient evaluation of the trainings for CHWs; and the potential for bias on the part of CHWs in conducting initial assessments and re-assessments following the parent workshops. The attachment assessment tool developed for this project is based on other validated assessment tools, including the Circle of Security Index [8], the Parent Development Index [9], and the Adult Attachment Interview [10]. The CHWs received extensive initial training on the use of the tool; however, it is acknowledged that CHWs are not skilled psychologists, and thus the validity and reliability of assessments in the field pose many challenges. Some of these challenges can be addressed by repeated opportunities for training and evaluation using videotaping of CHWs actually performing assessments. 

Although overall nutritional status was assessed—and noted to be positively correlated caregiver-child attachment—there was no ability to determine specific micronutrient deficiencies that may confound and thus impact caregiver-child attachment. Lozoff *et al.*, among others, has not only noted the association between iron deficiency anemia and cognitive performance and social-emotional development in young children, but has also recently shown that home-based interventions (above and beyond simple iron supplementation) to foster child development in children with iron deficiency anemia result in improved social-emotional scores [22]. The burden of anemia in children in this current project is unknown; estimates of iron deficiency anemia in children in Kenya vary widely, from as low as 7.4% to as high as 89%, depending on the specific geographic region and population under study [23,24,25]. It can be assumed, however, that given overall nutritional status, the burden of iron deficiency anemia (and likely other micronutrient deficiencies) is sufficient to impact childhood development, and thus caregiver-child attachment. The inability to determine the actual level of such micronutrient deficiencies and their impact on attachment is thus another limitation in this study.

The associations drawn between attachment and other measures (such as nutritional status) are based on cross-sectional data, and cannot describe cause and effect; there is no ability yet to contrast changes in attachment with changes in other measures of child health and development. In addition, this project does not suggest proof of a cause and effect relationship between provision of parent workshops and subsequent changes in the assessment of attachment, as there are many reasons why re-assessments might change. The potential for CHW (or “administrator”) bias in conducting the initial assessments (since they were familiar with the specific area of the slum communities) and re-assessments following interventions (since the CHW conducting the re-assessment was involved in the intervention) is acknowledged. While these limitations are noted, they are all considered as potential areas for program improvement and refinement, rather than as fundamental flaws or programmatic weaknesses that cannot be overcome. For example, future research could explore how best to balance the efforts to have community engagement (particularly in the selection of CHWs) with the need to improve objectivity and reduce the potential CHW biases noted above.

## 5. Conclusions

The primary contribution of this project is to provide a description of caregiver-child attachment in a sample of residents of Nairobi slums when derived from PHC visits. This project provides a demonstration of the potential in responding to the health and social needs of children in urban African slum settings in ways that are measurably effective, sustainable, at relatively low cost, and with active community participation. 

The emphasis on ECD is of special importance, as it is the potential point of intervention that may change the nature of the biological embedding of the social determinants of health that influences health outcomes in later life. In addition to familiar approaches to PHC, the project addresses particular health and social needs of children that seldom receive attention and yet are exceedingly important for their healthy development—namely caregiver-child attachments that are secure and loving, in contrast to insecure and neglectful attachments that can result in lifelong harmful effects. 

Although this project is modest in terms of its scope and coverage, it could serve as a focal point for bringing interested parties together in considering how these concepts and actions relating so importantly to ECD might be incorporated into more advanced and extensive work that could benefit children across all of sub-Saharan Africa.
